# Basal metabolic rate in Brazilian patients with type 2 diabetes: comparison between measured and estimated values

**DOI:** 10.20945/2359-3997000000103

**Published:** 2019-02-01

**Authors:** Thais Steemburgo, Camila Lazzari, Juliano Boufleur Farinha, Tatiana Pedroso de Paula, Luciana Vercoza Viana, Alvaro Reischak de Oliveira, Mirela Jobim de Azevedo

**Affiliations:** 1 Universidade Federal do Rio Grande do Sul Universidade Federal do Rio Grande do Sul Programa de Pós-Graduação em Alimentação, Nutrição e Saúde Porto Alegre RS Brasil Programa de Pós-Graduação em Alimentação, Nutrição e Saúde, Universidade Federal do Rio Grande do Sul (UFRGS), Porto Alegre, RS, Brasil; 2 Universidade Federal do Rio Grande do Sul Universidade Federal do Rio Grande do Sul Departamento de Nutrição Porto Alegre RS Brasil Departamento de Nutrição, Universidade Federal do Rio Grande do Sul (UFRGS), Porto Alegre, RS, Brasil; 3 Hospital de Clínicas de Porto Alegre Divisão de Endocrinologia Porto Alegre RS Brasil Divisão de Endocrinologia, Hospital de Clínicas de Porto Alegre, Porto Alegre, RS, Brasil; 4 Universidade Federal do Rio Grande do Sul Universidade Federal do Rio Grande do Sul Escola de Educação Física Porto Alegre RS Brasil Escola de Educação Física, Universidade Federal do Rio Grande do Sul (UFRGS), Porto Alegre, RS, Brasil

**Keywords:** Indirect calorimetry, basal metabolic rate, energy metabolism, type 2 diabetes

## Abstract

**Objectives:**

The aims of this study are to investigate which of the seven selected predictive equation for estimating basal metabolic rate (BMR) is the best alternative to indirect calorimetry (IC) and to evaluate the dietary energy intake in patients with type 2 diabetes.

**Subjects and methods:**

Twenty-one patients with type 2 diabetes participated in this diagnostic test study. Clinical and laboratorial variables were evaluated as well as body composition by absorptiometry dual X-ray emission (DXA) and BMR measured by IC and estimated by prediction equations. Dietary intake was evaluated by a quantitative food frequency questionnaire. Data were analyzed using Bland–Altman plots, paired *t-*tests, and Pearson's correlation coefficients.

**Results:**

Patients were 62 (48-70) years old, have had diabetes for 8 (2-36) yeas, and 52.4% were females. The mean body composition comprised a fat-free mass of 49.8 ± 9.4 kg and a fat mass of 28.3 ± 7.2 kg. The energy intake was 2134.3 ± 730.2 kcal/day and the BMR by IC was 1745 ± 315 kcal/day. There was a wide variation in the accuracy of BMR values predicted by equations when compared to IC BMR measurement. Harris-Benedict, Oxford, FAO/WHO/UNO equations produced the smallest differences to IC, with a general bias of < 8%. The FAO/WHO/UNO equation provided the best BMR prediction in comparison to measured BMR.

**Conclusion:**

In patients with type 2 diabetes, the equation of the FAO/WHO/UNO was the one closest to the BMR values as measured by IC.

## INTRODUCTION

Type 2 diabetes is the most common form of the diabetes mellitus (DM), usually occurs in adult life, and is associated with obesity in about 80% of cases. The primary strategy for treating obese patients with type 2 diabetes is the loss of body mass through changes in lifestyle, which has been associated with improvement in glycemic control (1). Among these interventions, an appropriate dietary prescription with the goal of reducing body weight, taking into account each patient's daily energy needs, is essential. The main energy requirement component is the total energy expenditure (TEE), and calculating the TEE requires knowledge of the basal metabolic rate (BMR) (2).

The most accurate procedure for measuring BMR is indirect calorimetry (IC), which is considered the reference method. However, its use is limited due to equipment costs, the need for qualified and trained personnel, and time constraints. In clinical practice, several predictive equations have been developed as alternative methods for estimating BMR (3–6).

The variability of BMR may depend on several factors, such as sex, ethnicity, age, physical activity, genetic factors, the presence of diabetes or obesity, body composition, and caloric intake (7). Several studies have evaluated BMR using prediction equations in different populations (8–10) and specific ethnic groups, as demonstrated in the US and Dutch (11), Belgian (12), Pima Indians (13), Caucasians (14) and Asian populations (15–18)

In nondiabetic individuals, different studies have demonstrated that the equations described by Harris-Benedict (3), Schofield (4), Mifflin-St. Jeor (5), and the FAO/WHO/UNO (6) can overestimate or underestimate the BMR value as compared with IC. A cross-sectional study in Brazilian eutrophic and obese men showed a variation between the prediction equations as compared to IC (8). The equation that was closest to the BMR was the one proposed by Mifflin-St. Jeor, with a difference of −9.1% in obese subjects and of 0.9% in eutrophic subjects (8). In 40 obese Brazilian women, the equations of Harris and Benedict and the FAO/WHO/UNO that use weight and height in their formulas were the only ones that did not present statistically significant differences when compared to IC (9). Finally, in another study conducted in 86 Spanish obese women also submitted to a dietary intervention, the most accurate formula for estimating BMR was the Mifflin-St. Jeor equation and the smallest bias was demonstrated by Owen's equation using IC as the reference method (10).

In patients with type 2 diabetes data on BMR prediction equations have been previously described (13,14,16–22), however, in Brazilian diabetic patients it was scarcely investigated (19). Indeed, one study that included only Brazilian obese women with type 2 diabetes, the equation of Mifflin-St. Jeor underestimated BMR in −2.6% and the FAO/WHO/UNO equation overestimated BMR in 10.6% as compared with IC (19). Two other studies proposed formulas for predicting BMR in diabetic patients, irrespective of sex (20,21). In a cross-sectional study conducted in 65 patients with type 2 diabetes, the inclusion of fasting blood glucose as a variable in a BMR predictive formula improved its predictability for real BMR by > 3% (20). Hyperglycemia, defined as fasting blood glucose > 180 mg/dL, increased the BMR by up to 8% (20). A retrospective analysis of severely obese patients was performed to compare the BMR of patients with and without diabetes (21). Obese Japanese patients with diabetes had a higher BMR than nondiabetic patients (18) and the presence of diabetes should be included as a variable in their proposed BMR predictive equation (21)). In fact, the association of poor glycemic control with a high BMR, as evaluated by high A1c values, has already been demonstrated (22). In addition, the reduction of BMR has been negatively associated with both endogenous and exogenous insulin values, as demonstrated in a study conducted in 58 patients with type 2 diabetes (23).

Given that most predictive equations overestimate BMR in obese subjects, that the majority of patients with diabetes are obese or overweight, and that hyperglycemia has been associated with an increase in BMR, it is important to evaluate the best prediction equations for calculating BMR in order to recommend an adequate dietary intervention for diabetic patients. In addition, studies in Brazilian diabetics are scarce, the aim of the present study was to evaluate which predictive BMR equation was the best alternative to IC in Brazilian patients with type 2 diabetes.

## SUBJECTS AND METHODS

### Study design and patients

This diagnostic study test was conducted in 21 patients with type 2 diabetes, defined as patients over 30 years of age at onset of diabetes, with no previous episode of ketoacidosis or documented ketonuria, and treatment with insulin only after 5 years of diagnosis. Patients attending the outpatient clinic of the Endocrine Division of the Hospital de Clínicas de Porto Alegre, Brazil were selected on the basis of the following criteria: did not receive dietary counseling from a registered dietitian during the previous 6 months, age < 80 years, serum creatinine < 2 mg/dL, normal liver and thyroid function tests, and absence of renal disease, cardiac failure, or any acute or consumptive disease. The study protocol was approved by the Ethics Commission of Hospital de Clínicas de Porto Alegre (number 15.0625) and all subjects signed a written consent form.

### Clinical evaluation

Patients were classified as current smokers or not. Physical activity status, according to activities during a typical day and based on the short version of the International Physical Questionnaire (IPAQ) (24) adapted to local habits (25), was classified into three levels: (1) low, (2) moderate, and (3) high. Blood pressure was measured twice to the nearest 2 mmHg, after a 10 minutes rest, using an Omron HEM-705CP digital sphygmomanometer (Omron Healthcare, Inc., Bamockburn, IL, USA). Hypertension was defined as blood pressure ≥ 140/90 mmHg measured on two occasions or the use of antihypertensive drugs (26). The body weight and height of patients (without shoes and coats) were obtained using a calibrated and anthropometric scale (Filizola^®^). Measurements were recorded to the nearest 100g for weight and to the nearest 0.1 cm for height. Body mass index (BMI) was calculated as weight in kilograms divided by the square of the height in meters. Body composition was assessed by dual-energy X-ray absorptiometry (Healthcare^®^ GE Medical Systems) for determining fat mass (kg) and fat-free mass (FFM) (kg). The procedure was performed in a specialized clinic by an imaging specialist.

### Dietary assessment

The usual diet was evaluated by the quantitative food frequency questionnaire (FFQ) previously validated for patients with diabetes which details 80 items divided into 10 food groups (27).

### Laboratory measurements

Blood samples were obtained after a 12-hour fasting. Plasma glucose level was determined by a glucose oxidase method, the A1c test by ion exchange high-performance liquid chromatography (Merck-Hitachi L-9100 glycated hemoglobin analyzer, reference range 4.7%-6.0%; Merck Diagnostica, Darmstadt, Germany), serum cholesterol and triglycerides by enzymatic colorimetric methods (Merck; Boeringher Mannheim, Buenos Aires, Argentina), and high-density lipoprotein (HDL) cholesterol by a homogeneous direct method (autoanalyzer, ADVIA 1650). Low-density lipoprotein (LDL) cholesterol was calculated using the Friedewald formula: LDL cholesterol = total cholesterol – HDL cholesterol – triglycerides/5.

### Basal metabolic rate measurement

The measurement of BMR was performed by IC. The IC protocol consisted of 10 min of rest on a gurney in dorsal decubitus, followed by 30 minutes of collection of exhaled gases using the canopy dilution technique and a coupled collection device. An open circuit calorimeter (QUARK RMR, Cosmed, Rome, Italy) was used for determining VO_2_ (oxygen consumption) and VCO_2_ (carbon dioxide production). To calibrate the equipment, the volume of the turbine flowmeter was first calibrated electronically by the system, followed by calibration of the collector plates using a known gas concentration. This process was repeated for each test to standardize the measurement. The first 10 min of gas collection were excluded from the analysis; thus, VO_2_ and VCO_2_ (L/min) obtained during the final 20 min of each collection (mean value of the period) were used for the calculation of BMR. The equation proposed by Weir (28) was used to obtain values in kcal/min, which does not require the use of protein metabolism by incorporating a correction factor: [(3.9 x VO_2_) + (1.1 x VCO_2_)]. The result in kcal/min was multiplied by 1,440 min to obtain the value for 24 hours. The subjects were asked not to perform any type of physical activity of moderate or high intensity during the 24 hours preceding the test, and not to consume alcohol or caffeine. The smoking patients were instructed not to smoke 12 hours before the day of BMR measurement. Additionally, the subjects were instructed to fast for 12 hours prior to the test, with only the *ad libitum* intake of water being permitted, and to have a good night's sleep of at least 8 hours. Finally, all subjects came to the test site using a motor vehicle to avoid energy expenditure before the determination of BMR. All tests were performed between 07:00 and 08:00 in a temperature-controlled (20 ºC to 25 ºC) and sound-controlled room under low luminosity. All medications in use were maintained during the study period and patients received their usual medication after the IC.

### Selection of equations for estimating basal metabolic rate (BMR)

Predictive equations for estimating BMR were selected by searching previous publications on this subject (8–21). To be included, the equations had to have been developed for adults, men, and women, and based on body weight, height, age, sex, and/or fat mass (FM). Equations derived only for specific ethnic groups or for subjects with BMI ≥ 40 kg/m^2^ were not included. In accordance with these criteria, we included a total of seven BMR equations (Supplement 1).

**Supplement 1:** Selected equations for estimating basal metabolic rate (BMR)

Reference	BMR predictive equations	Number of subjects	Sample characteristics	Weight/BMI
**Harris-Benedict** (3)				
Men	66 + [13.8 × W (kg)] + [5.0 × Ht (cm)] – [6.8 × (A)]			
Women	655 [9.5 × W (kg)] + [1.9 × Ht (cm)] – [4.7 × (A)]	–	–	–
**Schofield** (4)				
Men age < 60 years	[0.048 × W (kg) + 3.653] × 239	-	-	-
Men age > 60 years	[0.049 × W (kg) + 2.459] × 239			
Women age < 60 years	[0.034 × W (kg) + 3.538] × 239			
Women age > 60 years	[0.028 × W (kg) + 2.755] × 239			
**Mifflin-St. Jeor** (5)				
Men	[W (kg) × 10] + [Ht (cm) × 6.25] – [(A) × 5] + 5	251 men	Healthy adults	–
Women	[W (kg) × 10] + [Ht (cm) × 6.25] – [(A) × 5] + 5 −166	247 women		–
**FAO/WHO/UNO** (6)				
Men age < 60 years	11.6 × W (kg) + 879	7173		–
Men age > 60 years	13.5 × W (kg) + 487	men/women	Healthy adults	
Women < 60 years	8.7 × W (kg) + 829			
Women > 60 years	10.5 × W (kg) + 596			
**Gougeon and cols.** (20)		25 men	Patients with type 2 diabetes	100 ± 3 kg
Men and women	375 + (85 × W) – (48 × FM) + (63 × FPG)	40 women		37 ± 1 kg/m²
**Bernstein** (29)				
Men	(11.0 × W) + (10.2 × Ht) – (5.8 × A) – 1,032	48 men	Obese	
Women	(7.48 × W) + (0.42 × Ht) - (3.0 × A) + 844	154 women		
**Oxford** (30)		800 men		
Men 30-60 years	14.2 × weight + 593	5,000 women	Healthy adults	–
Men > 60 years	13.5 × weight + 514			
Women 30-60 years	9.74 × weight + 694			
Women > 60 years	10.1 × weight + 569			

BMI: body mass index; W: weight; A: age; Ht: height; FM: fat mass; FPG: fasting plasma glucose (mM)*.

* Unit of measure described in the original article of the equation (20)).

### Statistical analyses

The sample size calculation was based on a study conducted in obese and normal-weight subjects that evaluated the accuracy of predictive BMR equations for estimating energy expenditure as compared to IC (8). Assuming an alpha error of 5% and a power of 80%, a total of 21 patients with type 2 diabetes could be included.

The BMR was estimated by seven prediction equations commonly used according to sex and age: Harris-Benedict (5), Schofield (6), Mifflin-St. Jeor (7), FAO/WHO/UNO (8), Gougeon (20), Bernstein (29), and Oxford (30). We also evaluated the estimated caloric intake (27) versus measured BMR. The Shapiro-Wilk normality test was used to determine the distribution of the variables. The bias was calculated as follows: estimated BMR – measured BMR. For each prediction equation the percentage of deviation of estimated BMR from measured BMR was calculated: [(estimated BMR − measured BMR) / measured BMR] × 100. Estimated and measured BMR was compared by paired Student's *t*-test. The agreement between estimated and measured BMR was graphically examined by plotting the differences between the predicted and the measured BMR against their mean values, with 95% limits of agreement (mean difference ± 1.96 standard deviation of the difference) (31). Pearson's correlation coefficient was used to assess the correlation between estimated and measured BMR. Results are expressed as means and standard deviations, or medians (P25-P75). Data were analyzed using SPSS version 21.0, and Bland and Altman plot values were analyzed by the R Project for Statistical Computing software (version 3.3.3). A *p* value of < 0.05 was considered significant.

## RESULTS

Twenty-one patients with type 2 diabetes were evaluated [62 (48-70) years of age; 28.6% were older than 65 years old, 8 (2-36) years of diabetes duration and 52.4% of women]. Most patients in the study were sedentary (66.7%), and 90.5% were overweight/obese. The mean body composition comprised 49.8 ± 9.4 kg of fat-free mass and 28.3 ± 7.2 kg of fat mass. The mean total energy intake was 2134.3 ± 730.2 kcal/day. Also, the presence of hypertension was observed in all patients (100%). The lipid profile was within normal limits; however, the glycemic control expressed by fasting glucose and A1c test showed altered levels, as expected in diabetic patients. With regard to drug treatment, all patients used oral antihyperglycemic agents (100%) and antihypertensive agents (100%), and 38.1% (n = 8) used lipid-lowering agents. The characteristics of the sample are described in Table 1.

**Table 1:** Characteristics of patients with type 2 diabetes

Variable		n (21)
Age (years)		62 (48-70)
Duration of diabetes (years)		8 (2-36)
Sex (female)		11 (52.4%)
Psysical activity – level 1 (sedentarism) (%)		14 (66.7%)
Weight (kg)		85.2 ± 18.0
Height (cm)		168.3 ± 10.2
BMI (kg/m²)		29.4 (20.2-37.4)
Fat-free mass (kg)		49.8 ± 9.4
Fat mass (kg)		28.3 ± 7.2
Total energy intake (kcal/day)		2134.3 ± 730.2
Hypertension (%)		21 (100%)
Fasting plasma glucose (mg/dL)		155.6 ± 58.4
A1C test (%)		7.6 (6.1-10.0)
Total cholesterol (mg/dL)		166.4 ± 36.1
LDL cholesterol (mg/dL)		88.7 ± 32.8
HDL cholesterol (mg/dL)		47.2 ± 14.2
Triglycerides (mg/dL)		145 (49-342)
Medications		
	Oral antihyperglycemic	21 (100%)
	Antihypertensive agents	21 (100%)
	Hypolipidemic agents	8 (38.1%)

Data are presented as median (25th-75th), percentage (%) or mean ± standard deviation. BMI: body mass index; A1C: glycated hemoglobin; HDL: high-density lipoprotein; LDL: low-density lipoprotein.

Table 2 shows mean and standard deviation values of measured BMR and estimated BMR with selected predictive equations, bias (percentage deviation) and 95% limits of agreement. The variables assumed a normal distribution according to the Shapiro-Wilk test (data not shown). The mean of the BMR (kcal/day) verified by IC was statistically different by 1,745 ± 315 kcal/day kcal/day from the BMR values (kcal/day) estimated by the prediction equations. According to the percentage, the prediction equation that underestimated the measured BMR the most were those of Bernstein (-20.1%), followed by Mifflin-St Jeor (-13.6%) and Schofield (-11.7%). Also, the equations that in lower values underestimated the measured BMR were the Harris-Benedict (-7.8%) equation when using weight and height together and the Oxford equation (-7.6%) when using only weight. The value of overestimation (8.7%) observed corresponded to the equation reported by Gougeon and cols. (20) when including FM and fasting plasma glucose (FPG). The equation that most closely estimated the BMR measured was that of the FAO/WHO/UNO (-5.6%), which also uses only the weight in its formula.

**Table 2:** Evaluation of measured and estimated BMR in patients with type 2 diabetes

Variable		Mean	SD	95% Limits of Agreement^1^	p value
Measured BMR by IC (kcal/day)		1745	315		
Estimated BMR (kcal/day)					
**Harris-Benedict** (5)		1607.8	340.0		0.002†
	Bias^2^ (kcal/day)	-137.4		(-486.0; 211.1)	
	Percentage %^3^	-7.8			
**Schofield** (6)		1552.2	387.2		< 0.001†
	Bias^2^ (kcal/day)	-193.1		(-534.0; 147.8)	
	Percentage %^3^	-11.7			
**Mifflin-St.Jeor** (7)		1508.4	319.0		< 0.001†
	Bias^2^ (kcal/day)	-236.9		(-540.0; 66.2)	
	Percentage %^3^	-13.6			
**FAO/WHO/UNO** (8)		1647.1	331.1		0.012†
	Bias^2^ (kcal/day)	-98.2		(-416.1; 219.8)	
	Percentage %^3^	-5.6			
**Gougeon** (20)		1593.55	285.4		< 0.001†
	Bias^2^ (kcal/day)	151.8		(-206.5; 510.0)	
	Percentage %^3^	8.7			
**Bernstein** (29)		1376.7	224.3	(-801.2; 64.0)	< 0.001†
	Bias^2^ (kcal/day)	-368.6			
	Percentage %^3^	-21.1			
**Oxford** (30)		1615.1	353.6		0.002†
	Bias^2^ (kcal/day)	-130.2		(-459.5; 199.2)	
	Percentage %^3^	-7.5			

† Paired Student's *t*-test: to compared estimated and measured BMR

1 Mean difference ± 1.96 SD of the difference.

2 Estimated − measured (kcal in 24h.

3 Difference/measured) × 100 (%).

BMR: basal metabolic rate; IC: indirect calorimetry; SD: standard deviation.

**Figure 1:**
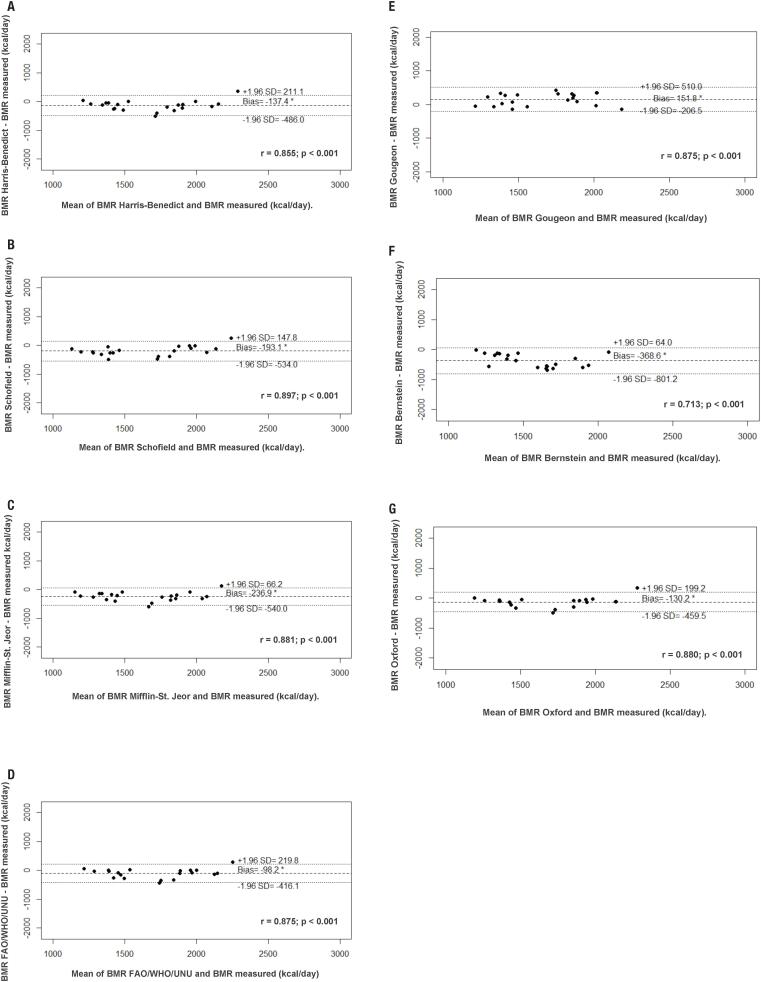
Bland and Altman plots comparing indirect calorimetry (IC) and the following prediction equations for basal metabolic rate (BMR) in patients with type 2 diabetes: A) Harris-Benedict (5); B) Schofield (6); C) Mifflin-St. Jeor (7); D) FAO/WHO/UNO (8); E) Gougeon and cols. (20); F) Bernstein (31), and G) Oxford (30)

When we evaluated energy intake by FFQ *vs.* the BMR by CI, was observed a difference of approximately 400 kcal/day (2134.3 ± 730.2 vs. 1745 ± 315 kcal/day, respectively), which corresponds to an overestimation of 23%. The Bland and Altman plots for the difference between predicted and measured BMR against the mean obtained equations are reported in Figure 1. The graphs demonstrating the agreement between the values of measured and estimated BMR suggest a poor correlation between the two methods, with wide limits of agreement. However, strong positive correlations were observed (p < 0.001) between methods. The correlation shows the association between dependent and independent variables, and BMR was significantly positively correlated (p < 0.01) with fat-free mass (FFM) (r = 0.859), BMI (r = 0.593), sex (r = 0.281), and age (r = 0.509). There were no significant correlations between BMR fat mass, FPG or between BMR and A1 test.

## DISCUSSION

There is little research that compares the BMR measured by IC with that estimated by prediction equations in patients with type 2 diabetes. Our study shows a wide range of differences between predicted and measured BMR. The Harris-Benedict (when using weight and height together), and the Oxford and FAO/WHO/UNO equations (when using only weight), showed mean differences between measurements and predicted BMR < 8%. These results suggest that these three equations are the most suitable equations to estimate BMR in patients with diabetes. However, the equation that presented lower values of bias (-98.2 kcal) and difference (-5.6%) compared to BMR measured by IC was that described by FAO/WHO/UNO. This finding is in agreement with a previous study conducted in women with obesity, but without diabetes, which also demonstrated an underestimation of the formulas proposed by the FAO/WHO/UNO and Harris-Benedict (9). In obese women and men with diabetes, these equations showed an overestimation compared to BMR measured by IC (19,20). Validation studies show that the FAO weight and height equation is the most accurate (32), which is in agreement with our results. An explanation for this could be that this equation derived from a similar ethnic population as found in Brazil, which is mainly composed of a population of European descendants, especially Italians.

The Harris-Benedict equation is one of the most commonly used equations in clinical practice and, as it is the oldest, has undergone the most extensive validation (3). A Belgian study which validated BMR equations in 536 women with a wide variety of body weight (18.5-50 kg/m^2^) showed that the Harris-Benedict equation and the Mifflin-St. Jeor equation are a reliable tools to predict BMR (12).

The Mifflin-St. Jeor equation was developed with a large sample of obese subjects. Several studies proposed this equation as the most valid to estimate BMR in overweight and obese subjects aged 19-69 years (78% accurate predictions) (10,12). In our study, the Mifflin-St. Jeor equation presented a bias of −236 kcal, underestimating by −13.6% the value of the BMR measured. In the same way, we observed that the equations proposed by Schofield and Bernstein presented high values of −193.1 and −368.6 kcal, underestimating the BMR value measured by IC by −11.7% and 20.1%, respectively.

Although the Bland and Altman plots revealed a poor agreement between the equations and the IC, it is important to emphasize the correlations found between the methods. However, the correlation indicates only how the two methods interact linearly by not correctly expressing the agreement between them.

The findings of this study are clinically relevant and suggest that the best equation for estimating BMR in patients with type 2 diabetes is the one that considers the current weight. We observed a positive correlation of the BMR with BMI (r = 0.593, p = 0.005). This result is in agreement with the study conducted in Brazilian obese women with type 2 diabetes (19). These outcomes corroborate the importance of using the weight as an independent variable in the prediction equations to correctly estimate the BMR of patients with diabetes, since the equations selected in this study included weight as independent variable (3–6,20,29,30). It is important to more accurately estimate the BMR of patients with type 2 diabetes with overweight and obesity to promote specific and individualized dietary management for these patients.

There is scarce scientific evidence indicating how the presence of diabetes may influence basal metabolism. However, some authors have confirmed higher BMR values in subjects with type 2 diabetes compared with controls without the disease (18,20,29,30). Another factor to consider is the association between diabetes and excessive body weight, since obese people have both increased FM and FFM fat mass and fat-free mass, which contributes to the increase in BMR (33).

In patients with type 2 diabetes evaluated in this study, we observed a strong correlation of BMR measured with FFM (r = 0.859; p < 0.001). No correlation was found between BMR and FM. In agreement with other studies, we noted that the inclusion of body composition (FM and/or FFM) into the equations did not improve the accuracy of BMR prediction (10–12). This is a relevant finding because equations based on anthropometric parameters (weight and height) are more feasible in clinical practice than are body composition-based equations.

Gougeon and cols. (20) evaluated the BMR of women with type 2 diabetes, proposing an equation to predict the BMR that tested plasma glucose, and FM as some of its independent variables, which justifies a better fit in the model equation. However, when we evaluated, the specific equation for patients with type 2 diabetes, described by Gougeon, using FM and fasting glycemia, it showed an 8.7% overestimation of the measured BMR.

In our study, we observed an overestimation of 23% of calorie consumption, which corresponds to around 400 kcal/day. These results suggest that in this group, patients consumed more calories than is actually required for their basal metabolism, and this explains the difficulty of weight loss in patients with obesity and diabetes. Moreover, regardless of the formula used, actual intake is higher than measured intake and estimations. The difference between the equations (-368.6 to −98.2 kcal/day) shows that with the diet prescription, any of the equations would lead to an adequate weight loss.

Our findings indicate that in the absence of a gold standard method the best equation for estimating BMR in patients with type 2 diabetes is the equation reported by the FAO/WHO/UNO. This equation presented a smaller value of bias, around −5.6%, and for clinical practice this corresponds to 100 kcal/day less when we apply the formula in this group of patients. It is important to remember that this same group overestimated their calorie intake by 400 kcal/day.

The present study is limited in particular by the small sample size and it is also possible to observe a different BMR behavior with regard to sex in the distribution of our group of diabetic patients in the Bland and Altman plots. In fact, sex is a variable that appear to influence BMR. The decreased BMR with increasing age, specifically in women, may also be a result of changes in body composition caused by menopause (34). Also, from the age of 20, women present a reduction of BMR by about 2% per decade, and the decrease of the FFM directly influences this decline (35,36). Thus, for a better evaluation in the comparison of the real BMR and the BMR calculated by prediction equations, we suggest the importance of a study conducted in patients with type 2 diabetes using a larger sample in order to evaluate the difference between the sex with their inherent characteristics.

In conclusion, this study showed that there is a great variation in the accuracy of BMR prediction equations. The accuracy of BMR prediction equations should be adequate to promote the efficacy of dietary counseling and treatment of diabetes. The findings showed that among the selected prediction equations, the BMR estimated by the FAO/WHO/UNO equation was the closest to the measured BMR as assessed by the percentage deviation.
